# Endophytic Bacteria in Toxic South African Plants: Identification, Phylogeny and Possible Involvement in Gousiekte

**DOI:** 10.1371/journal.pone.0019265

**Published:** 2011-04-26

**Authors:** Brecht Verstraete, Daan Van Elst, Hester Steyn, Braam Van Wyk, Benny Lemaire, Erik Smets, Steven Dessein

**Affiliations:** 1 Laboratory of Plant Systematics, Katholieke Universiteit Leuven, Leuven, Belgium; 2 Laboratory of Plant Growth and Development, University of Antwerp, Antwerp, Belgium; 3 South African National Biodiversity Institute, Pretoria, South Africa; 4 HGWJ Schweickerdt Herbarium, University of Pretoria, Pretoria, South Africa; 5 Netherlands Centre for Biodiversity Naturalis, Leiden University, Leiden, The Netherlands; 6 National Botanic Garden of Belgium, Meise, Belgium; University of Birmingham, United Kingdom

## Abstract

**Background:**

South African plant species of the genera *Fadogia*, *Pavetta* and *Vangueria* (all belonging to Rubiaceae) are known to cause gousiekte (literally ‘quick disease’), a fatal cardiotoxicosis of ruminants characterised by acute heart failure four to eight weeks after ingestion. Noteworthy is that all these plants harbour endophytes in their leaves: nodulating bacteria in specialized nodules in *Pavetta* and non-nodulating bacteria in the intercellular spaces between mesophyll cells in *Fadogia* and *Vangueria*.

**Principal Findings:**

Isolation and analyses of these endophytes reveal the presence of *Burkholderia* bacteria in all the plant species implicated in gousiekte. Although the nodulating and non-nodulating bacteria belong to the same genus, they are phylogenetically not closely related and even fall in different bacterial clades. *Pavetta harborii* and *Pavetta schumanniana* have their own specific endophyte – *Candidatus* Burkholderia harborii and *Candidatus* Burkholderia schumanniana – while the non-nodulating bacteria found in the other gousiekte-inducing plants show high similarity to *Burkholderia caledonica*. In this group, the bacteria are host specific at population level. Investigation of gousiekte-inducing plants from other African countries resulted in the discovery of the same endophytes. Several other plants of the genera *Afrocanthium*, *Canthium*, *Keetia*, *Psydrax*, *Pygmaeothamnus* and *Pyrostria* were tested and were found to lack bacterial endophytes.

**Conclusions:**

The discovery and identification of *Burkholderia* bacteria in gousiekte-inducing plants open new perspectives and opportunities for research not only into the cause of this economically important disease, but also into the evolution and functional significance of bacterial endosymbiosis in Rubiaceae. Other South African Rubiaceae that grow in the same area as the gousiekte-inducing plants were found to lack bacterial endophytes which suggests a link between bacteria and gousiekte. The same bacteria are consistently found in gousiekte-inducing plants from different regions indicating that these plants will also be toxic to ruminants in other African countries.

## Introduction

Some species of Rubiaceae from South Africa are known to cause a disease of domestic ruminants called gousiekte, an Afrikaans name literally translated as ‘quick disease’. Gousiekte is one of the six most important plant poisonings in southern Africa. In 2008, the expected annual impact of mortalities from gousiekte on the livestock industry in South Africa was estimated at approximately R9 million in the case of cattle, and R5.2 million in the case of small stock [1]. Hitherto research into gousiekte was primarily carried out by veterinarians and focused on the aetiology and pathology of the disease. More recently toxicologists and chemists showed interest in the disease resulting in the isolation and chemical characterization of pavettamine, the compound claimed to be the cause of the cardiotoxicosis [2]–[4]. Gousiekte is a disease of ruminants characterized by acute heart failure without early warning signs 4–8 weeks after the initial ingestion of certain bacteriophilous Rubiaceae. *Vangueria pygmaea* (syn. *Pachystigma pygmaeum*) is the most important of these plants, followed in descending order of importance by *Fadogia homblei*, *Pavetta harborii*, *Vangueria thamnus* (syn. *Pachystigma thamnus*), *Pavetta schumanniana* and *Vangueria latifolia* (syn. *Pachystigma latifolia*) [5].

Significantly all gousiekte-inducing plants belong to Rubiaceae or coffee family, the fourth most species-rich flowering plant family with more than 13,000 species comprising about 611 genera [6]. It is a predominantly tropical and subtropical family but representatives occur on all continents, except Antarctica. To date gousiekte has, with one exception (a case of African buffalo poisoning in Zimbabwe [7]), only been diagnosed in the northeastern part of South Africa and this coincides with the distribution of the toxic plants: the geographical ranges of all six gousiekte-inducing plants overlap in the former Transvaal region [5]. *P. harborii*, *V. latifolia* and *V. thamnus* are even restricted to that region, while *F. homblei*, *P. schumanniana* and *V. pygmaea* have a distribution range that extends further north indicating that gousiekte may in fact occur in other countries, but has not been diagnosed yet [8]. A wide variety of growth forms is present in Rubiaceae: woody shrubs are most common but small herbs, lianas and large rainforest trees are also encountered. Five of the gousiekte-inducing plants are geofructices (*F. homblei*, *P. harborii*, *V. latifolia*, *V. pygmaea* and *V. thamnus*) and one is a woody shrub or small tree (*P. schumanniana*). Geofructices are plants with extensive or massive woody, perennial, underground stems and this enables the plants to sprout before the grass starts growing in spring, or in late summer when the grass withers [9]. During these two periods the green aboveground foliage and twigs of geofructices are readily consumed in large amounts by grazing livestock such as cattle and sheep [10].

The two *Pavetta* species that are involved in gousiekte have distinct bacterial nodules in their leaves. This type of leaf endosymbiosis is a rare and intimate interaction between plants and bacteria. Amongst flowering plants, Rubiaceae has the largest number of species with bacterial leaf nodules, these structures being present in three, distantly related genera, namely *Pavetta*, *Psychotria* and *Sericanthe* [11]. In *Psychotria*, the symbiotic cycle was shown to be obligate and cyclic: presence of the bacterial partner is required for host survival and the endophyte is retained within the host plant during all stages of its life cycle [12]. In the case of bacterial leaf nodules all previous attempts to identify the microbial symbiont based on morphological characterization have failed because its establishment in culture has not been successful as yet [13]. Recent molecular studies based on sequence analysis of 16S rDNA revealed that endophytes in *Pavetta* and *Psychotria* are host specific and belong to *Burkholderia*, a genus of highly diverse and adaptive proteobacteria [13]–[15].

A possible link between bacteria and gousiekte was postulated by Van Wyk et al. [16] following the discovery of bacterial symbionts in gousiekte-inducing members of *Fadogia* and *Vangueria*. Endophytic bacteria can be defined as bacteria that colonize the internal tissues of the plant without the host showing external signs of infection or other negative effects [17]. One possible advantage to bacteria in colonizing the internal tissues of a host plant is the presence of a uniform and protective environment. In return the endophyte can be beneficial to its host by promoting plant growth or by preventing infection by phytopathogenic organisms [18].

Before we can answer the question whether these endophytic bacteria and their host plant collaborate in causing gousiekte, further fundamental knowledge about occurrence and identity of the endophytes is essential. Our principal objective is not to give a conclusive causality but to identify the bacterial endophytes and to elucidate their phylogenetic context. Adding several related Rubiaceae species that grow in the same area will help determine whether endophytic bacteria are limited to the gousiekte-inducing plants. If endophytic bacteria are only found in the species that cause gousiekte it might indicate a possible involvement of the bacteria in gousiekte. It should be noted however, that a definitive causation cannot be proved from association studies alone. Nevertheless, identification of the different actors is the first step in finding a possible remediation of gousiekte.

## Results

Using a cultivation-independent approach, bacterial endophytes are found in all of the six gousiekte-inducing Rubiaceae species. The identification of the bacteria was performed using the standard method of comparing the sequence similarity of the 16S rDNA region [19]. Additional support for the identity is obtained through a combined phylogenetic analysis of the three molecular markers 16S, *gyrB* and *recA*.

The two species of *Pavetta* involved in gousiekte both have visible bacterial nodules in their leaves. The endophyte of *P. schumanniana* specimens from D.R.Congo and Zambia have already been identified and described as *Candidatus* Burkholderia schumanniana [18]. All DNA sequences of the endophytes in South African *P. schumanniana* are identical to the Congolese and Zambian ones and are thus considered as the same species. Several clones were obtained for the 16S rDNA region of *P. harborii* and only one unique sequence emerged, which indicates the presence of only one bacterium species. Different replicas of *P. harborii* were tested and the bacterial 16S rDNA sequences for all specimens are identical. BLAST analysis of the 16S rDNA sequences of the endophytes confirmed the bacterial identity as *Burkholderia*. It is closely related to *Candidatus* Burkholderia schumanniana and their 16S rDNA sequences have a difference of 1%.

Leaves of *F. homblei*, *V. latifolia*, *V. pygmaea* and *V. thamnus* do not have dark spots (nodules) on their leaf blades. Cultivation-independent analysis using sequences of 16S rDNA revealed the presence of non-nodulating endophytic bacteria in these species. The cloning experiments resulted in several clones of all four species indicating that only one endophyte per plant species is present. Several replicas from different geographic locations were tested for all species. Gousiekte plants originating from other African countries (D.R.Congo and Zambia) harbour the same bacteria as the South African specimens. The 16S rDNA regions of the endophytes of the four gousiekte-inducing species are 99.9% homologous and BLAST searches revealed that the sequences are almost identical (99.9%) to *Burkholderia caledonica*.

**Figure 1:**
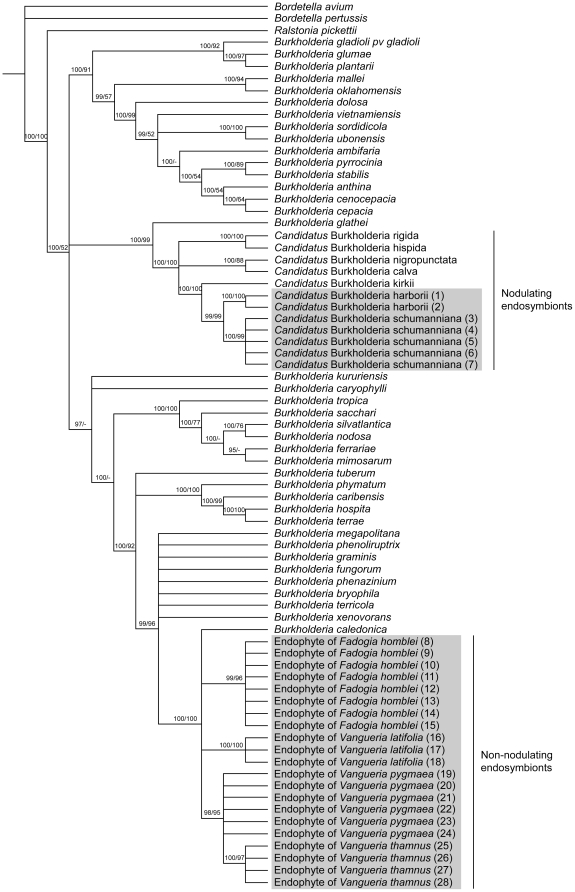
Phylogenetic relationships within the bacterial genus *Burkholderia.* The tree is based on 16S rDNA, *gyrB* and *recA* data. Numbers on branches represent Bayesian posterior probabilities and maximum parsimony bootstrap support. Gousiekte-inducing plants are indicated in grey boxes. Numbers between brackets correspond to the numbers in Table S1.

Besides 16S rDNA, two widely used housekeeping genes (*gyrB* and *recA*) were added for phylogenetic analysis. Because all 16S clones from the cloning experiments are identical, only one sequence per plant specimen was used to facilitate the phylogenetic analysis. The different geographic replicas were retained in the analysis. The combined dataset of the three genetic markers comprised the sequences of three outgroup species, 38 *Burkholderia* species, five nodulating endophytes and the endophytes of 28 gousiekte-inducing plants. Replicates of the bacteria found in *P. harborii* are identical and are related to *Candidatus* Burkholderia schumanniana, the endophyte of the gousiekte-inducing plant *P. schumanniana*. The non-nodulating endophytes of *F. homblei*, *V. latifolia*, *V. pygmaea* and *V. thamnus* cluster together with *B. caledonica* in a strongly supported clade (Figure 1). Although there is almost no variation in the 16S rDNA sequences, the endophytes of the individual gousiekte-inducing species group together because of minimal differences in *gyrB* and *recA* sequences.

In one of the species, *F. homblei*, we succeeded in isolating and cultivating the endophytic bacteria. The isolation procedures on leaves yielded only one bacterium species. These isolates were proven to be closely related to *B. caledonica* by the DNA analysis and they fall in the same clade as the endophytes identified by the cultivation-independent analysis (Figure 1). Both types of culture medium (LB and PCAT) gave consistent results.

Twenty-four Rubiaceae plants, which grow in the same area as the gousiekte-inducing plants but are not implicated in the disease, were investigated for the presence of bacterial endophytes. They belong to 14 species in the genera *Afrocanthium*, *Canthium*, *Keetia*, *Psydrax*, *Pygmaeothamnus* and *Pyrostria* that are part of Vanguerieae, the tribe containing four of the gousiekte-inducing plants. There are no visual bacterial nodules present in the leaves of any of the specimens investigated. Despite different biological and technical replicas, none of the species is shown to have endophytic bacteria inside their leaves.

## Discussion

Nodulating bacterial leaf endosymbiosis has been the subject of several scientific studies resulting in the identification of the endophytes as members of the genus *Burkholderia* [16]–[18]. This genus of β-proteobacteria has also been found to nodulate the plant roots of legumes [20]. The endophyte of the gousiekte plant *P. schumanniana* has already been identified as *Candidatus* Burkholderia schumanniana and is proven to be host specific [18].

We showed that the nodulating endophyte of *P. harborii* is closely related to *Candidatus* Burkholderia schumanniana (Figure 1), but the DNA sequences are slightly different. Based on the 99% 16S rDNA sequence similarity to delineate bacterial species [19] and because the endophytes of other *Pavetta* species are host specific [18], we conclude that the endophyte in *P. harborii* is a new species. *P. schumanniana* and *P. harborii* are morphologically closely related, hence the close similarity of their endophytes is to be expected. *P. harborii*, however, is a geofructex, whereas *P. schumanniana* is a proper shrub or small tree; their geographical ranges are also mutually exclusive. Cultivation of the endophytes of *Pavetta* has not been successful yet [16] and because uncultured organisms should be recorded under the provisional status *Candidatus* [21], we propose the name ‘*Candidatus* Burkholderia harborii’ for this taxon.

This is the first molecular investigation of the endophytes of gousiekte plants and it provides unequivocal evidence that members of *Fadogia* and *Vangueria*, unlike in the case of *Pavetta,* do not have visible bacterial nodules; instead there are non-nodulating bacteria present in the leaves. This confirms earlier observations based on light and electron microscopy [11].

The 16S rDNA sequence similarity between the non-nodulating bacteria found in four gousiekte plants and the nodulating endophytes of the two *Pavetta* species is about 96%, which indicates that they are not closely related. This conclusion is also reached when observing the relationships on the phylogenetic tree, which is based on three molecular markers (Figure 1). The nodulating and non-nodulating endophytes belong to the same genus *Burkholderia*, but they fall into different well-supported clades. This indicates that both lineages of bacteria independently developed the strategy to incorporate themselves in the leaves of Rubiaceae species. The only difference is that the endophytes of *Pavetta* and *Psychotria* are confined to discrete nodules (visible to the naked eye), while the endophytes of *Fadogia* and *Vangueria* remain diffusely distributed in the intercellular spaces among cells in the leaf tissues [11]. The phylogenetic tree indicates that the non-nodulating endophytes in *Fadogia* and *Vangueria* are very similar to *Burkholderia caledonica* (Figure 1). In fact the 16S rDNA sequences are 99.9% identical. Several clones per plant species and specimens from different geographic locations gave consistent results.

*B. caledonica* is a soil bacterium previously isolated from the rhizosphere of grapevine (*Vitis* sp.) and sugarcane (*Saccharum officinarum*) [22]. Noteworthy is that some of the closely related *Burkholderia* species are endophytes themselves (e.g. *B. bryophila* is an endosymbiont of moss, [23]) while others are soil bacteria that are in close contact with the root systems of plants (e.g. *B. xenovorans* is found in the rhizosphere of the coffee plant, [24]).

Although the 16S rDNA regions are identical, the *gyrB* and *recA* sequences reveal that the endophytes of each plant species form separate clades, indicating host specificity of the bacteria at population level. This means that different populations of the same bacterial species are found in different plant species. Based on this, it can be hypothesized that the interaction between bacteria and plants in this lineage of Rubiaceae recently evolved and that the bacteria are still undergoing speciation.

Until now all attempts to cultivate the nodulating *Burkholderia* of *Psychotria* have been unsuccessful [16]. In the present study, however, we were able to grow the non-nodulating endophyte of *F. homblei* on agar plates. Only one bacterium species emerged and its identification corroborated the results of the cultivation-independent analysis. Significantly, one of the tested *F. homblei* plants was grown from seeds collected in the wild. It has been suggested that nodulating bacteria in species of *Psychotria* could similarly be transferred to the next plant generation through the seeds [15]. The fact that our cultivated *F. homblei* has the same endophyte as the wild specimens may point to the presence of bacteria in the seeds. This would explain the occurrence of distinct populations of endophytes in the different gousiekte-inducing plants as shown by the phylogenetic analysis (Figure 1).

**Figure 2:**
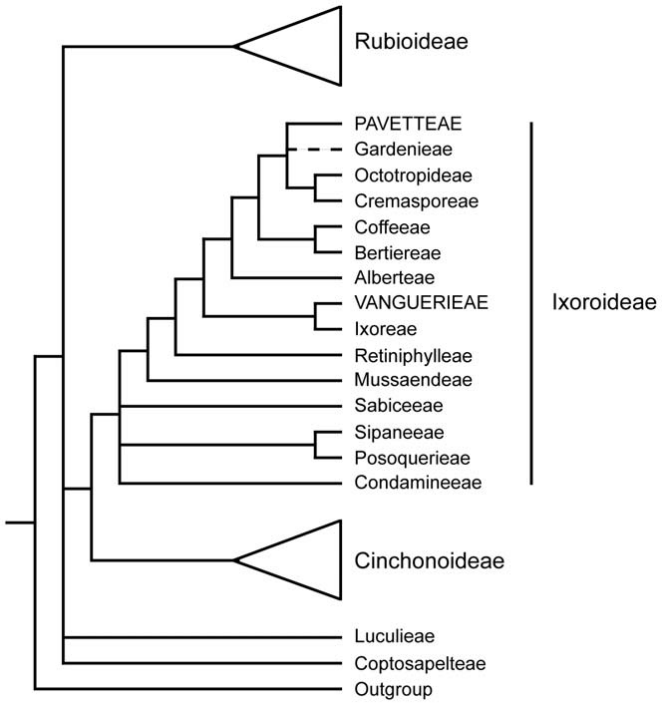
Adapted phylogenetic tree of the plant family Rubiaceae [36]. The gousiekte-inducing plants are part of the subfamily Ixoroideae, one of the three subfamilies in Rubiaceae. Nevertheless, they are not closely related as they belong to different tribes: *Fadogia* and *Vangueria* belong to Vanguerieae, while *Pavetta* belongs to Pavetteae. The respective tribes are indicated in capital letters.

Since almost a century, veterinarians have searched for the cause of gousiekte and this resulted in the denotation of six toxic plants: *F. homblei*, *V. latifolia*, *V. pygmaea*, *V. thamnus*, *P. harborii* and *P. schumanniana* [5]. We already pointed out that they all of these belong to Rubiaceae, more in particular to the subfamily Ixoroideae. Within the subfamily the species are not closely related: *Fadogia* and *Vangueria* are two genera of the tribe Vanguerieae, while *Pavetta* is a member of the tribe Pavetteae (Figure 2). Endophytic bacteria of the genus *Burkholderia* contained in specialized nodules are already known for the Pavetteae but this study is the first to establish the identity of the non-nodulating bacteria in the Vanguerieae. In South Africa many toxic plants are found causing a wide range of plant poisonings and mycotoxicoses [8], but why only six plants are responsible for gousiekte remains enigmatic. It is likely that other toxic rubiaceous plants are ignored in areas where gousiekte plants have already been identified [11]. To investigate whether the endophytic bacteria are limited to the gousiekte plants we added 24 specimens to our sampling (Table S2). These belong to 14 species that overlap in distribution area with the gousiekte-inducing plants and that are abundantly present. The genera involved are *Afrocanthium*, *Canthium*, *Keetia*, *Psydrax*, *Pygmaeothamnus* and *Pyrostria* and these are all part of Vanguerieae, the tribe containing four of the gousiekte-inducing plants. We could not detect visible bacterial nodules in their leaves and after careful molecular investigation, no endophytic bacteria could be found. *Pygmaeothamnus zeyheri* and *P. chamaedendrum* are very similar to *V. pygmaea* and *V. thamnus* and they often occur next to each other in the field. We tested these two plants several times and no bacterial DNA was amplified. Particularly noteworthy is that these two plants were tested for gousiekte by South African veterinarians but were found to be non-toxic [25]. These findings could support a possible role of the bacteria in causing gousiekte.

Geographically, gousiekte occurs in the northeastern part of South Africa and most of the outbreaks happen in the former Transvaal region. Nevertheless, it is presumed that it may in fact occur in other countries [8]. Only very recently, gousiekte has been diagnosed in wild African buffalo from Zimbabwe after eating *P. schumanniana* [7]. This indicates a wider distribution for the disease and it also shows that not only domestic livestock can be affected. In our study we included some gousiekte plants from D.R.Congo and Zambia to test whether the presence of endophytic bacteria is geographically limited. This is not the case since we consistently found the same bacteria in plants from different regions. If *Burkholderia* bacteria play a role in causing gousiekte, it is highly probable that the same plants growing in other African countries will also be toxic to ruminants.

The discovery and identification of *Burkholderia* bacteria in all investigated gousiekte-inducing plants open new perspectives and opportunities for research into the cause of the disease. A question that still remains unanswered is the origin of the putative toxin: are the plants or the endophytic *Burkholderia*, or perhaps both together, responsible for the production of pavettamine? It is well known that bacterial endosymbionts can be important in the production of toxic metabolites in plants [26]–[27]. It has, for example, been shown that endosymbiotic *Burkholderia* bacteria produce rhizoxin, a phytotoxin, in members of the plant pathogenic fungus *Rhizopus* [28]. Ongoing research into the cultured endophytes of *F. homblei* holds great promise in answering this question and thus giving perspective in a possible remediation of gousiekte.

In summary, toxic gousiekte-inducing plants are shown to have nodulating or non-nodulating *Burkholderia* bacteria. Host plant specificity at population level indicates a recent interaction and predicts ongoing speciation. Other South African Rubiaceae that grow in the same area as the gousiekte-inducing plants were tested and found to lack bacterial endophytes which suggests a link between bacteria and gousiekte. Discovery of the same endophytes in non South African gousiekte-inducing plants points to a wider occurrence of the disease. Our results not only shed new light on the evolution of bacterial endosymbiosis in Rubiaceae but also open new perspectives for further research into the functional significance of this phenomenon in the family, as well as its possible involvement in the formation of the gousiekte-causing toxin.

### Description of ‘*Candidatus* Burkholderia harborii’

‘*Candidatus* Burkholderia harborii’ (harborii, from the specific epithet of the host plant, *Pavetta harborii*, which was named after its discoverer Cyril Cecil Harbor (1883-1940)). [(*ß-Proteobacteria*, genus *Burkholderia*); NC; G-; R; NAS (GenBank accession numbers JF265202, JF265179, JF265225), oligonucleotide sequence complementary to unique region of 16S rDNA 5′-TCTGTTAAGACCGGTGTGAAATCCCTGGGCTC-3′, oligonucleotide sequence complementary to unique region of *gyrB* gene 5′-TACGGAGAACCGCGGCACTGAGGTGCACTTCC-3′, oligonucleotide sequence complementary to unique region of *recA* gene 5′-CACGCTGCAGGTGATTGCTGAGATGCAGAAGC-3′; S (*Pavetta harborii*, leaf)]. Verstraete et al., this study.

## Materials and Methods

Most of the plant material was collected during a field expedition in South Africa but additional samples were obtained from the National Botanic Garden of Belgium and the South African National Biodiversity Institute. Detailed information on the six gousiekte-inducing species and their respective bacterial endophyte can be found in Table S1. Herbarium vouchers for the other Rubiaceae species that are investigated in this study are mentioned in Table S2.

Before extraction of the bacterial DNA the silica-dried leaves were rinsed using 70% ethanol to avoid bacterial contamination. Extraction of the DNA was performed using the E.Z.N.A.^TM^ HP Plant DNA Mini Kit (Omega Bio-Tek).

Initially, PCR amplification of bacterial 16S rDNA was done with universal primers 16SB and 16SE [17]. A second more specific reverse primer 16S2 was also used to avoid amplification of chloroplast homologues [18]. For the amplification of DNA gyrase, subunit B (*gyrB*) and recombinase A (*recA*), primers were used as proposed by Spilker et al. [29]. The polymerase chain reactions (PCR) were run on a GeneAmp PCR System 9700 (Applied Biosystems, Foster City, California, USA) under a temperature profile of 94°C for 2 min followed by 30 cycles of 94°C for 45 sec, 55°C (16S rDNA) or 58°C (*gyrB* and *recA*) for 60 sec, and 72°C for 90 sec. The PCR products of 16S rDNA were ligated into pGEM-T vector (Promega), according to the manufacturer's instructions, and transformed into JM109 *E. coli* by heat shock. Plasmid purification was obtained by using a PureYield^TM^ Plasmid Miniprep System (Promega). Purified plasmid products were sent to Macrogen for sequencing (Macrogen Inc, Seoul, Korea).

The sequences were assembled and edited using Geneious 5.3 [30]. All new DNA data are deposited in GenBank and the accession numbers can be found in Table S1. Related bacterial sequences of *Burkholderia* were obtained from the BCCM/LMG Bacteria Collection (Belgian Co-ordinated Collections of Micro-organisms/Laboratory of Microbiology, Ghent University, http://bccm.belspo.be) and GenBank. Detailed information on these sequences can be found in the supplementary table S1 of Lemaire et al. [18]. A preliminary sequence alignment was performed in Geneious using a plugin for Muscle [31] followed by manual adjustments resulting in an unequivocal alignment. Phylogenetic trees were estimated using Bayesian Inference and Maximum Parsimony. Bayesian analysis was inferred using MrBayes 3.1 [32], running four Markov chains sampling every 100 generations for three million generations. A general time reversible model of DNA substitution with gamma-distributed rate variation across invariant sites was used (GTR+I+G). This model was chosen by performing hierarchical likelihood-ratio tests in MrModeltest v.3.06 [33]. Maximum parsimony analyses were conducted using Paup* v.4.0b10a [34]. Heuristic searches were conducted with TBR branch swapping on 10,000 random addition replicates, with five trees held at each step. Non-parametric bootstrap analysis was carried out to calculate the relative support for individual clades found in the parsimony analysis. For each of 1,000 bootstrap replicates, a heuristic search was conducted with identical settings as in the original heuristic analysis.

For growing the endophytic bacteria of *F. homblei* in culture, young leaves were collected. Leaf surfaces were sterilized with 80% ethanol for 5 min followed by 10 min in sodium hypochlorite (1%) and finally washed with sterile distilled water. Sterility was checked by placing sterilized leaf fragments in liquid LB medium. For endophyte extraction, sterile leaves were crushed in autoclaved mortars with 0.85% sodium chloride as buffer. The resulting fluid was spread on LB and PCAT agar plates [35]. Single colonies were picked out and grown in liquid medium for DNA analysis. Bacterial DNA was extracted using the DNeasy Blood & Tissue kit (Qiagen GmbH). Amplification and sequencing of the selected bacterial DNA markers was carried out as stated above.

## Supporting Information

Table S1 **Detailed information on the endophytes of the six gousiekte-inducing plants.** List of the investigated endophytes with origin, host plant voucher specimen and GenBank accession numbers. Herbarium vouchers are deposited at BR or PRE and acquisition numbers refer to the living collection of the National Botanic Garden of Belgium (NBGB). The endophytes cultivated on agar plates are indicated with an asterisk.
(DOC)

Table S2 **Detailed information on the investigated Rubiaceae plants that are not linked with gousiekte.** None of the here listed specimens has endophytic bacteria inside their leaves.
(DOC)
