# Correction: Endophytic Bacteria in Toxic South African Plants: Identification, Phylogeny and Possible Involvement in Gousiekte

**DOI:** 10.1371/annotation/e7fa3cdf-c7bc-439f-83a2-36da63ef6c08

**Published:** 2011-06-24

**Authors:** Brecht Verstraete, Daan Van Elst, Hester Steyn, Braam Van Wyk, Benny Lemaire, Erik Smets, Steven Dessein

The wrong journal was given for references 13, 14, 15, and 23. The correct references are:

13. Van Oevelen S, De Wachter R, Robbrecht E, Prinsen E (2004) 'Candidatus Burkholderia calva' and 'Candidatus Burkholderia nigropunctata' as leaf gall endosymbionts of African Psychotria. Int J Syst Evol Microbiol 54: 2237-2239.

14. Van Oevelen S, De Wachter R, Vandamme P, Robbrecht E, Prinsen E (2002) Identification of the bacterial endosymbionts in leaf galls of Psychotria (Rubiaceae, angiosperms) and proposal of 'Candidatus Burkholderia kirkii' sp. nov. Int J Syst Evol Microbiol 52: 2023-2027.

15. Lemaire B, Van Oevelen S, De Block P, Verstraete B, Smets E, et al. (2011) Identification of the bacterial endosymbionts in leaf nodules of Pavetta (Rubiaceae). Int J Syst Evol Microbiol doi:ijs.0.028019-0.

23. Vandamme PA, Opelt K, Knöchel N, Berg D, Schönmann S, et al. (2007) Burkholderia bryophila sp. nov. and Burkholderia megapolitana sp. nov., moss-associated species with antifungal and plant-growth-promoting properties. Int J Syst Evol Microbiol 57: 2228-2235.

