# Supplementary material for: Endophytic Bacteria in Toxic South African Plants: Identification, Phylogeny and Possible Involvement in Gousiekte
Source: PLoS One. 2011 Apr 26;6(4):e19265. doi: 10.1371/journal.pone.0019265 (PMC3082559; doi:10.1371/journal.pone.0019265)
Supplement: Table S1 — Detailed information on the endophytes of the six gousiekte-inducing plants. List of the investigated endophytes with origin, host plant voucher specimen and GenBank accession numbers. Herbarium vouchers are deposited at BR or PRE and acquisition numbers refer to the living collection of the National Botanic Garden of Belgium (NBGB). The endophytes cultivated on agar plates are indicated with an asterisk. (DOC) [file pone.0019265.s001.doc]

| **N** | **Taxon** | **Origin** | **Host plant voucher** | | **GenBank accession number** | | |
| --- | --- | --- | --- | --- | --- | --- | --- |
|  |  |  |  |  | 16S | *gyrB* | *recA* |
| 1 | *Candidatus* Burkholderia harborii | South Africa | Lemaire & Verstraete 49A | BR | JF265202 | JF265179 | JF265225 |
| 2 | *Candidatus* Burkholderia harborii | South Africa | Lemaire & Verstraete 49B | BR | JF265203 | JF265180 | JF265226 |
| 3 | *Candidatus* Burkholderia schumanniana | D.R.Congo | 20001942-57 | NBGB | HQ849126 | HQ849235 | HQ849182 |
| 4 | *Candidatus* Burkholderia schumanniana | Zambia | 20041430-66 | NBGB | HQ849127 | HQ849236 | HQ849183 |
| 5 | *Candidatus* Burkholderia schumanniana | Zambia | Dessein et al. 1099 | BR | HQ849124 | HQ849233 | HQ849180 |
| 6 | *Candidatus* Burkholderia schumanniana | Zambia | Dessein et al. 1137 | BR | HQ849125 | HQ849234 | HQ849181 |
| 7 | *Candidatus* Burkholderia schumanniana | South Africa | Lemaire & Verstraete 1 | BR | HQ849128 | HQ849237 | HQ849184 |
| 8 | Endophyte of *Fadogia homblei** | South Africa | 20101674-73 | NBGB | JF265201 | JF265178 | JF265224 |
| 9 | Endophyte of *Fadogia homblei* | South Africa | Lemaire & Verstraete 3 | BR | JF265197 | JF265174 | JF265220 |
| 10 | Endophyte of *Fadogia homblei* | South Africa | Lemaire & Verstraete 9 | BR | JF265200 | JF265177 | JF265223 |
| 11 | Endophyte of *Fadogia homblei** | South Africa | Lemaire & Verstraete 22 | BR | JF265194 | JF265171 | JF265217 |
| 12 | Endophyte of *Fadogia homblei* | South Africa | Lemaire & Verstraete 30 | BR | JF265196 | JF265173 | JF265219 |
| 13 | Endophyte of *Fadogia homblei* | South Africa | Lemaire & Verstraete 50 | BR | JF265198 | JF265175 | JF265221 |
| 14 | Endophyte of *Fadogia homblei* | South Africa | Lemaire & Verstraete 57 | BR | JF265199 | JF265176 | JF265222 |
| 15 | Endophyte of *Fadogia homblei* | South Africa | Lemaire & Verstraete 292 | BR | JF265195 | JF265172 | JF265218 |
| 16 | Endophyte of *Vangueria latifolia* | South Africa | Lemaire & Verstraete 69 | BR | JF265205 | JF265182 | JF265228 |
| 17 | Endophyte of *Vangueria latifolia* | South Africa | Lemaire & Verstraete 74 | BR | JF265206 | JF265183 | JF265229 |
| 18 | Endophyte of *Vangueria latifolia* | South Africa | Lemaire & Verstraete 141 | BR | JF265204 | JF265181 | JF265227 |
| 19 | Endophyte of *Vangueria pygmaea* | Zambia | Dessein et al. 666 | BR | JF265211 | JF265188 | JF265234 |
| 20 | Endophyte of *Vangueria pygmaea* | Zambia | Dessein et al. 726 | BR | JF265212 | JF265189 | JF265235 |
| 21 | Endophyte of *Vangueria pygmaea* | South Africa | Lemaire & Verstraete 26A | BR | JF265207 | JF265184 | JF265230 |
| 22 | Endophyte of *Vangueria pygmaea* | South Africa | Lemaire & Verstraete 26B | BR | JF265208 | JF265185 | JF265231 |
| 23 | Endophyte of *Vangueria pygmaea* | South Africa | Lemaire & Verstraete 28 | BR | JF265209 | JF265186 | JF265232 |
| 24 | Endophyte of *Vangueria pygmaea* | South Africa | Lemaire & Verstraete 42 | BR | JF265210 | JF265187 | JF265233 |
| 25 | Endophyte of *Vangueria thamnus* | South Africa | Bester 10538 | PRE | JF265213 | JF265190 | JF265236 |
| 26 | Endophyte of *Vangueria thamnus* | South Africa | Lemaire & Verstraete 25A | BR | JF265214 | JF265191 | JF265237 |
| 27 | Endophyte of *Vangueria thamnus* | South Africa | Lemaire & Verstraete 25B | BR | JF265215 | JF265192 | JF265238 |
| 28 | Endophyte of *Vangueria thamnus* | South Africa | Steyn 1835 | PRE | JF265216 | JF265193 | JF265239 |
