# Supplementary material for: Endophytic Bacteria in Toxic South African Plants: Identification, Phylogeny and Possible Involvement in Gousiekte
Source: PLoS One. 2011 Apr 26;6(4):e19265. doi: 10.1371/journal.pone.0019265 (PMC3082559; doi:10.1371/journal.pone.0019265)
Supplement: Table S2 — Detailed information on the investigated Rubiaceae plants that are not linked with gousiekte. None of the here listed specimens has endophytic bacteria inside their leaves. (DOC) [file pone.0019265.s002.doc]

| **Taxon** | **Origin** | **Voucher** | |
| --- | --- | --- | --- |
| *Afrocanthium gilfillanii* (N.E.Br.) Lantz | South Africa | Lemaire & Verstraete 32 | BR |
| *Afrocanthium mundianum* (Cham. & Schltdl.) Lantz | South Africa | Lemaire & Verstraete 33 | BR |
| *Afrocanthium mundianum* (Cham. & Schltdl.) Lantz | South Africa | Lemaire & Verstraete 84 | BR |
| *Canthium ciliatum* (D.Dietr.) Kuntze | South Africa | Lemaire & Verstraete 122 | BR |
| *Canthium ciliatum* (D.Dietr.) Kuntze | South Africa | Lemaire & Verstraete 281 | BR |
| *Canthium inerme* (L.f.) Kuntze | South Africa | Lemaire & Verstraete 235 | BR |
| *Canthium kuntzeanum* Bridson | South Africa | Lemaire & Verstraete 241 | BR |
| *Canthium kuntzeanum* Bridson | South Africa | Lemaire & Verstraete 250 | BR |
| *Canthium spinosum* (Klotzsch ex Eckl. & Zeyh.) Kuntze | South Africa | Lemaire & Verstraete 187 | BR |
| *Keetia gueinzii* (Sond.) Bridson | South Africa | Lemaire & Verstraete 97 | BR |
| *Keetia gueinzii* (Sond.) Bridson | South Africa | Lemaire & Verstraete 127 | BR |
| *Keetia gueinzii* (Sond.) Bridson | South Africa | Lemaire & Verstraete 210 | BR |
| *Keetia gueinzii* (Sond.) Bridson | South Africa | Lemaire & Verstraete 225 | BR |
| *Keetia gueinzii* (Sond.) Bridson | South Africa | Lemaire & Verstraete 300 | BR |
| *Psydrax fragrantissima* (K.Schum.) Bridson | South Africa | Lemaire & Verstraete 163 | BR |
| *Psydrax livida* (Hiern) Bridson | South Africa | Lemaire & Verstraete 14 | BR |
| *Psydrax livida* (Hiern) Bridson | South Africa | Lemaire & Verstraete 261 | BR |
| *Psydrax locuples* (K.Schum.) Bridson | South Africa | Lemaire & Verstraete 287 | BR |
| *Psydrax obovata* (Klotzsch ex Eckl. & Zeyh.) Bridson | South Africa | Lemaire & Verstraete 188 | BR |
| *Pygmaeothamnus zeyheri* (Sond.) Robyns | South Africa | Lemaire & Verstraete 2 | BR |
| *Pygmaeothamnus zeyheri* (Sond.) Robyns | South Africa | Lemaire & Verstraete 58 | BR |
| *Pygmaeothamnus chamaedendrum* (Kuntze) Robyns | South Africa | Lemaire & Verstraete 142 | BR |
| *Pyrostria hystrix* (Bremek.) Bridson | South Africa | Lemaire & Verstraete 92 | BR |
| *Pyrostria hystrix* (Bremek.) Bridson | South Africa | Lemaire & Verstraete 270 | BR |
